# Use of Peer Mentoring, Interdisciplinary Collaboration, and Archival Datasets for Engaging Undergraduates in Publishable Research

**DOI:** 10.3389/fpsyg.2019.00096

**Published:** 2019-02-06

**Authors:** Jonathan J. Hammersley, Micheal L. Waters, Kristy M. Keefe

**Affiliations:** Department of Psychology, Western Illinois University, Macomb, IL, United States

**Keywords:** undergraduate research, clinical psychology, archival data, collaboration, liberal arts college, state university, Interdisciplinary

We agree wholeheartedly with Dr. Sharon Brehm, the 2007 President of the American Psychological Association, who stated: “***I believe that undergraduate research is one of the three most valuable experiences that colleges and universities can offer their undergraduate students*** (Keynote Address, 24th Annual Mid-America Undergraduate Psychology Research Conference).” We would add that engaging in undergraduate research can be enjoyable and rewarding, for students as well as their faculty mentors. There is nothing quite like observing students becoming interested and engaged in research, planning and carrying their own projects, getting excited to analyze their data, and then experiencing the pride of presenting or publishing their project. This is perhaps one of the best aspects of being a psychology faculty member.

Teaching at large state universities, or at small liberal arts colleges, comes with certain challenges for conducting research with undergraduates. Other authors have addressed models for involving undergraduates in high-quality research and encouraging presentation and publication of findings(e.g., Gibson et al., 1996; Hughes, 2014; McKelvie and Standing, 2018). A plethora of research literature also addresses challenges of improving writing (Stellmack et al., 2012; Jorgensen and Marek, 2013; Greenberg, 2015) and statistical skills (e.g., Lyle and Crawford, 2011; Lim et al., 2015; Hartnett, 2016); or for a truly novel approach to teaching statistics, see Irving (2015). Other practices, such as fostering interest in quality research during introductory psychology courses, are also beyond the scope of our article.

Our article focuses on challenges inherent in engaging undergraduate students in high quality, publishable research at underfunded colleges and universities, which often have fewer resources dedicated to conducting research (lack of time, participants, equipment, and other support) and whose faculty have high teaching loads and service commitments. We also focus on the difficulties of researching certain topics and describe some potential solutions that could include collaborative efforts and utilization of archival data.

## Challenges

### Engaging Students

Engaging undergraduates in publishable research projects is challenging. Despite many clear benefits to both students and faculty (Landrum and Nelsen, 2002; Hughes, 2014; Woodzicka et al., 2015), a relatively low percentage of students take advantage of opportunities to conduct research. In the National Survey of Student Engagement through the Center for Postsecondary Research at Indiana University (National Survey of Student Engagement, 2018), only 21% of college seniors at public universities and 25% at private universities reported engaging in research with faculty. This number varies across institutions, such as Research I universities (26%), schools with fewer than 2,500 total students (30%), and with Arts & Sciences focus (44%), perhaps partly due to different definitions of research activities. Certain students (non-traditional seniors age 25+, 14%; first generation college students, 20%) report less involvement in research.

Thus, fostering student interest in faculty research programs is a significant challenge.

We have advertised research studies and the potential for undergraduate involvement within and outside of the department. Student abstracts from conference presentations are also displayed prominently within the department, so students are aware of research achievements of other students. Our labs have been fortunate in that the topics that we study seem to stimulate student interest and involvement: suicidality, depressive and anxiety symptoms, attentional deficits, traumatic experiences, mental health treatments, and drug use, for example.

### Clinical Research

Clinical and counseling psychology graduate program admissions are popular aspirations for undergraduates, and as the largest subspecialties in psychology, faculty can expect students to inquire about gaining experience researching clinical topics (Norcross et al., 2014). Further, students with aspirations for doctoral study in clinical or counseling psychology Ph.D. programs can expect requirements to include laboratory courses and research experience, strong letters of recommendations, and well-crafted personal statements, as well as high GPA and GRE scores (Norcross and Sayette, 2014).

However, it can also be very challenging to establish a clinical research program, especially at universities without access to psychiatric clients, that are not attached to teaching hospitals or clinics. Below we offer several further recommendations for establishing interesting research programs that can involve students.

## Recommendations

### Peer Mentoring

Clinical research and other interesting subtopics can be beneficial for recruiting student researchers potentially interested in becoming involved in publishable research. We just concluded a project on caffeine and nicotine use that involved many undergraduates and is likely to result in publications, and we recently began a multi-institutional collaborative project examining video game imagery, that has seemed popular among undergraduates looking for research experience.

One way we achieve undergraduate involvement is through the use of vertical peer supervision within labs, in which graduate students or more advanced undergraduate students can help supervise and mentor small teams of undergraduates. Undergraduates may relate to and feel less intimidated by fellow students, and may feel more comfortable asking questions or discussing mistakes or become more engaged in the research when noticing the enthusiasm of graduate students.

### Forming Research Collaborations

There are a number of advantages of interdisciplinary research collaboration as well as a few potential drawbacks. For example, interdisciplinary research teams tend to produce research that receives more citations and is thus influential (Wuchty et al., 2007). Scientific research, especially certain STEM and medical fields, appears to have become more collaborative in recent decades (Wuchty et al., 2007; Burroughs, 2017). Interdisciplinary collaboration can be complicated, and there may be risks involved, especially for early career, tenure-track faculty (Rhoten and Andrew, 2004; Moore et al., 2018); however, complex problems such as poverty, violence, and human behaviors or social issues may be best approached by diverse interdisciplinary research teams who bring a broad range of skills and knowledge (Gehlert et al., 2014; Graesser et al., 2018). Collaborative research can result in synergy (Katz and Martin, 1997) as well as increased creativity, motivation, and deeper, more nuanced understanding for students (Woodzicka et al., 2015). Interdisciplinary problem-based learning, a collaborative group learning process, developed to prompt students in health professions to learn beyond rote memory and to develop critical thinking, problem-solving, and research skills, which are associated with enhanced cognitive outcomes as well as student satisfaction, engagement, and perceived usefulness (Davidson and Major, 2014).

Moreover, as a result of the Job Outlook 2018 survey, (National Association of Colleges Employers, 2017) recently found that the popular skills that current employers now value included abilities in problem-solving (82.9%), work in a team (82.9%), written communication (80.3%), leadership (72.6%), analytical/quantitative areas (67.5%), and verbal communication (67.5%). Some faculty (Szostak, 2007; Everett, 2016) have suggested that these findings may speak to the importance of interdisciplinary training and research, to assist students in developing such skills.

In our experiences, collaborating across institutions and disciplines is an effective way to pool funds and resources such as equipment, supplies, and research assistants. For example, successful completion of interdisciplinary projects in collaboration with biology and chemistry departments, which allowed faculty and students from these disciplines to work together and learn about new areas of science and research from one another, demonstrate the potential of such arrangements. One project involving health effects of consuming alkalized watered, which was carried out between the psychology and chemistry departments at a small liberal arts college, allowed faculty and students to work together across disciplines and learn about one another's respective disciplines and research methodology. Several students were able to use the project for senior seminar capstone projects and presented the results on campus.

In another project carried out in a collaboration between psychology and biology departments at Western Illinois University, we are examining neurotransmitter genotypes related to addictive behaviors, from DNA obtained from saliva. Portions of this project have been presented both on campus and at major psychological conferences, and are also currently being written up for publication by faculty and students. Our labs have also collaborated on several occasions with other labs within the university and at other state universities to pool efforts and resources, resulting in a number of conference presentations and manuscript submissions. Between 5 and 10 students each year from 2014 through 2018 were also able to obtain excellent research experience and training from these endeavors.

Although interdisciplinary writing and research groups have been utilized at the graduate postgraduate levels (Cuthbert et al., 2009), a relative dearth of research literature exists on interdisciplinary research teams in undergraduate psychology. Models do exist for interdisciplinary integration of undergraduate psychology coursework at both liberal arts and Research I institutions (Golding and Kraemer, 2000; Ebersole and Kelty-Stephen, 2017), which might help students and faculty across departments appreciate and value concepts and scientific methods from other disciplines. Other faculty have also created courses with interdisciplinary assignments and teaching techniques (Ross et al., 2013) or “cluster courses” that revolve around an interdisciplinary topic (Wingert et al., 2014).

Burroughs (2017) recommends utilizing the expertise of librarians to help set up collaborative relationships, or searching for departments and individuals on campus who have shown a propensity for collaborative research. At Western Illinois University, our Center for Innovation in Teaching and Research has a searchable database of faculty research topics which can also be used to set up potential collaborations (http://www.wiu.edu/CITR/services/research.php).

### Archival Data

Another way in which we have successfully developed research programs that incorporate students is to utilize archival, or publicly available, databases to examine clinical topics. A primary benefit of accessing and analyzing archival data has been the study of topics that otherwise would not be possible (or would be very difficult) outside of medical schools or Research I universities.

In addition to studying clinical topics, especially behaviors with relatively low base rates (e.g., suicidality, inhalant abuse) or treatment outcomes that would otherwise take years to complete, additional benefits of archival data analysis might include the study of behaviors and epidemiology in large, nationally representative samples across genders, sexual orientation, or socioeconomics, that also allow for the use of statistics that require large sample sizes (e.g., structural equation modeling, moderation, and mediation analysis). In our lab, we have benefited from learning and refining new statistical approaches. Some behaviors we have been able to study would also have been difficult for a small college Institutional Review Board to review and approve.

While there can be many benefits to using archival data, several possible drawbacks also exist. For example, available archival data is often several years to decades old, and data collected through surveys often (though not always) preclude experimental designs and require correlational analyses. The available data from surveys or clinical ratings may not be a direct measurement of a behavior. Moreover, the datasets are often very complicated and can take weeks or months to clean and organize. Two archival datasets we have utilized for research projects (American College Health Association, 2009; Center for Collegiate Mental Health, 2015) required a lengthy application and review process, similar to a grant or journal submission. We have also used data from the SAHMSA Treatment Episodes Dataset (Substance Abuse Mental Health Services Administration, 2005), the Carolina Abecedarian Project (ABC; Campbell and Ramey, 2010), and mandated, publicly reported crime statistics on university websites for clinically relevant projects. Other colleagues have used Amazon's Mechanical Turk (MTurk; http://www.mturk.com; reviewed by Shapiro et al., 2013), the Institute for Social Research (https://www.icpsr.umich.edu/icpsrweb/), the Henry Murray Archive (https://murray.harvard.edu/), or the Midlife Development in the U.S. Study (MIDUS; http://midus.wisc.edu/) and have collected data through Reddit, Facebook, or other social media. Psi Chi has also been moving toward crowdsourcing and data sharing.

## Conclusion

We strongly encourage engaging undergraduates in faculty research programs, so that both students and faculty can experience the satisfaction and enjoyment that result from this collaboration. However, involving undergraduate students in quality research projects, especially clinical research that involves examining psychopathology or addiction, can be quite challenging. Strategically implementing procedures that include vertical peer mentoring, collaborating with colleagues across department/disciplines or institutions, and utilizing available archival databases can help faculty from all subdisciplines overcome some of the challenges. All in all, such procedures can be useful and allow for interesting and rewarding experiences while increasing the likelihood of publishable undergraduate research.

## Author Contributions

JJH developed the initial concept for the article, wrote the initial draft and revisions and was the primary author. MLW contributed to drafts of the article, reviewed and provided critical revisions, and created the reference list. KMK helped develop the initial concepts for the article and provided critical revisions. All authors approved the paper for submission.

### Conflict of Interest Statement

The authors declare that the research was conducted in the absence of any commercial or financial relationships that could be construed as a potential conflict of interest.
